# Use of the World Wide Web to Implement Clinical Practice Guidelines: A Feasibility Study

**DOI:** 10.2196/jmir.5.2.e12

**Published:** 2003-06-13

**Authors:** Jean-Gabriel Jeannot, Frédy Scherer, Valérie Pittet, Bernard Burnand, John-Paul Vader

**Affiliations:** ^1^University of LausanneInstitute of Social and Preventive MedicineSwitzerland; ^2^Centre Electronique de GestionMedical InformaticsSwitzerland

**Keywords:** Practice guidelines, Internet, decision support systems, clinical, appropriateness of care, quality of health care, back pain, laminectomy, endoscopy

## Abstract

**Background:**

Important efforts have been invested in the past few years in the development of quality clinical guidelines. However, the means for the effective dissemination of guidelines to practicing physicians have not been determined. Several studies have examined the possibilities offered by the World Wide Web (the Web), but studies examining the implementation of clinical guidelines in actual practice are clearly lacking.

**Objective:**

This study assessed the potential of the Web to implement clinical practice guidelines in actual clinical settings. It also documents the obstacles perceived by the physicians in their use of guidelines on the Internet to determine the role that the Web can play in the implementation of guidelines in practice.

**Methods:**

Two guidelines were developed using a standardized panel method and made available via the Web. One concerned indications for low-back surgery and the other dealt with indications for upper and lower digestive endoscopies. To identify obstacles to their use in clinical practice, 20 physicians were asked to consult the guidelines during consultations with patients. Answers were collected using 3 different questionnaires.

**Results:**

Questionnaires were completed for consultations involving 213 patients. Less than 50% of the physicians have direct access to the Internet in their examination room. For 75%, the use of the guidelines was easy and the time required to consult them acceptable (3.4 minutes on average, or 12% of the time spent with the patient). The fear that use of such guidelines might interfere with the physician-patient relationship was mentioned as a reason for not consulting the guidelines for 27 consultations. Taking into account their experience with the Web, 75% of the physicians considered that the Web has a great or very-great potential for the dissemination of guidelines and 78% indicated that they would use such guidelines if they became generally available for clinical questions that concerned them. Only 3 physicians had consulted guidelines on the Web prior to this study.

**Conclusions:**

The acceptance of use of clinical practice guidelines via the Web is high. The main limits to further use of such Web-based guidelines seem to be the lack of a computer connection in the physician's office or examining room and the fear that use of such guidelines might interfere with the physician-patient relationship. Though most participants appreciate the considerable potential of the Web for disseminating guidelines, only a small handful regularly use guidelines available on the Web. There are still numerous obstacles to the regular use of guidelines in clinical practice, some related to the physicians, others to the guidelines themselves.

## Introduction

This study assessed the potential of the World Wide Web to implement clinical practice guidelines in real clinical settings. It highlights the obstacles perceived by the physicians in their use of guidelines on the Internet.

### Dissemination of Guidelines Alone is Not Enough, it Needs to be Combined With an Implementation Strategy

Clinical practice guidelines are defined as systematically-developed statements to assist patient and practitioner in decisions about appropriate health care for specific clinical circumstances [1]. Clinical practice guidelines are intended to increase the quality of patient care by reducing variations in practice and to control costs through more-efficient use of health care resources [1]. But formulating guidelines is easier than making them work [2]. More than 50 systematic reviews on strategies and approaches for implementing guidelines in clinical practice have been undertaken in the last decade [3]. The results are, however, not straightforward. Strategies effective in one study were not effective in others. Even when a strategy was effective, it was often not clear what had caused the change [4]. A combination of different activities in a well-designed implementation plan is usually the most-effective approach [5,6]. Evidence-based medicine should be complemented by evidence-based implementation [5].

### The benefits of the Internet in Health Care Will Depend on its Ability to Provide Efficient and Effective Ways to Access and Use the Knowledge That We Need, When We Need It, and In the Right Format

A growing number of papers in the medical literature present information systems in general and on the World Wide Web in particular as a promising media to implement guidelines [7,8,9,10]. In spite of these enthusiastic opinions, proofs of the effectiveness of the Internet to implement guidelines are still lacking [11,12,13]. Several studies based their conclusions more on hopes than on strong evidence [14,15]. The more-interesting studies [16] have aimed at testing clinical guidelines that could be delivered over the Internet. Those authors conclude that when tested in clinical scenarios compliance of the physicians is better with electronic guidelines than with paper guidelines.

The aim of our study was to go one step further in assessing the potential of the Web to implement clinical practice guidelines in the physician's office in real clinical settings. The importance of validating the effectiveness of guidelines via the Web in clinical situations has been emphasized by several authors [17,18,12].

Some obstacles can be expected in terms of the difficulty of changing physician habits [19,20,21] and the perceived intrusion of the computer into the doctor-patient relation [22]. However, it is precisely because such resistance exists that studies to address ways to overcome that resistance are important. Before a strategy to implement change is selected the obstacles to change have to be identified.

## Methods

### Guidelines

The guidelines used in this study were developed using a standardized panel method (RAND) [23,24]. The proposed guidelines are designed to provide guidance for the individual patient and feedback to the physician, both of which are elements that have been identified as favorably impacting the successful implementation of clinical guidelines [25,26,27]. They consist of explicit criteria for the evaluation of the appropriateness of medical procedures, which combine a detailed review of the literature with systematically-developed collective-expert opinion. The concept of appropriateness refers to the relative weight of the benefits and harms of a medical or surgical intervention. An appropriate procedure is one in which "the expected health benefit exceeds the negative consequences by a sufficiently wide margin that the procedure is worth doing, exclusive of cost" [24]. The rationale behind the method is that randomized clinical trials, the gold standard for evidence-based medicine, often are not available or cannot provide evidence at a level of detail sufficient to apply to the wide range of patients seen in everyday clinical practice. The RAND method combines the best available scientific evidence with the collective judgment of experts to yield an assessment of the appropriateness of performing a procedure at the level of patient-specific symptoms, medical history, and test results.

The guidelines studied in this paper concerned the indications for low-back surgery (laminectomy) and upper and lower digestive endoscopy. They were then transcribed into HTML (Hyper Text Mark-up Language) and made available on the Web.

See Multimedia Appendix 1: PowerPoint presentation of laminectomy guideline (3 minutes).

### Participating Physicians

An invitation to participate in this study was sent to 98 physicians in private practice in the French-speaking part of Switzerland. They were chosen because of expressed interest in this feasibility study and because they were believed to have patients concerned about the subject of the 2 guidelines. They were informed that an inclusion criterion was an Internet connection. The general practitioners were asked to test both guidelines, the neurosurgeons and rheumatologists the low-back surgery guidelines (laminectomy) [28], and the gastroenterologists the endoscopy guidelines [29].

### Survey

The participating physicians first reported on their use of the Internet and about their computer equipment. Then, each participating physician was requested to use the electronic guidelines in the evaluation of all eligible patients during a period of 3 weeks, or a maximum of 20 patients. Any patient presenting with upper or lower gastrointestinal symptoms or with low-back pain or sciatica was eligible for inclusion in the study. For each eligible patient, the physician was asked to report on whether he/she consulted the Web guidelines, reasons for nonconsultation, length of consultation (total patient and online access to guidelines), difficulties in accessing or understanding the Web guidelines, appropriateness of the procedure, whether the procedure was proposed to the patient, and whether the patient would undergo the procedure.

At the end of this testing phase, the physician was asked about the acceptability of the Web site and ways to render it more accessible, acceptable, and user friendly. Questions centered on obstacles to use, functions which were particularly helpful or not used, functions that could be added, ease of use, usefulness, perceived potential (with improvement), and whether the use of the guidelines disturbed the physician in his/her work routine or in his/her relationship with his/her patient.

## Results

Of the 98 physicians, 33 manifested interest in participation. Of those 33, 20 (14 general practitioners, 1 gastroenterologist, 1 neurosurgeon, and 4 rheumatologists) consulted the guidelines for at least 1 patient. The main reasons for nonparticipation were lack of time and/or the unavailability of an Internet connection at the time of the study. The guidelines were consulted for 213 patients.

### Computer Equipment and Previous Experience With the Web

The response rate was 98% for the general items dealing with use of the Web and computer equipment. All 20 physicians had experience navigating on the Internet; 18 stated doing so at least once a week. The majority (13) indicated accessing both medical and non-medical sites, but only 3 were aware of guidelines available on the Web. None were aware of the National Guideline Clearinghouse Web site [30].

Though it would seem to be a requisite condition for our study, only 9 participants actually had a computer in their office, 5 have one in their secretariat, and 1 in another room in the practice. Concerning Internet connections, 8 physicians used an analog modem at 56 Kb/sec, 8 a digital modem (ISDN - Integrated Services Digital Network), and 4 a more-rapid connection.

See Appendix 2: questions and summary of responses for questionnaire "Computer equipment and previous experience with the Web (extract)."

### Use of the Guidelines Web Sites

For the whole set of questions, the average rate of response was 85%. The physicians consulted the back-surgery guidelines 104 times and the endoscopy guidelines 80 times. The main reasons for not consulting the guidelines were fear of disturbing the physician-patient relationship (n = 27) and that the situation was so clear that reference to guidelines was not necessary (n = 22).

In 87% of the cases, the computer was already turned on when the physician intended to consult the guidelines and in 94% of the cases the Internet connection was established without difficulty.

In 96% of the cases the physician was able to readily reply to the questions on the guidelines site. On average, the total length of the consultation was 27 minutes, including an average of 3.4 minutes consulting the guidelines site online (12% of total consultation time, range 3% to 33%).

According to the guidelines, the procedure was appropriate in 32% of the cases, uncertain in 14%, and inappropriate in 54%. In 90% of the cases the physician was in agreement with the treatment approach proposed by the guidelines.

See Appendix 3: questions and summary of responses for questionnaire "Use of guidelines sites."

### Evaluation of Guidelines

The response rate for the questionnaire concerning the general evaluation of the use of the two sites was 92%. Among the 20 physicians who had used both sites, 7 preferred the endoscopy site, qualifying it as more useful; 3 preferred the back-surgery site, qualifying it as faster. Almost all participants considered access to both sites as easy.

Nineteen felt the access time for both sites was acceptable.

Fifteen felt that the use of such guidelines as a decision tool was easy or very easy.

All felt that the use of the guidelines had little or no effect on their relationship with the patient. Fourteen stated that the use of the guidelines did not significantly disturb their working routine. Fourteen felt that such guidelines are of little or no use for determining the appropriateness of guidelines for medical procedures.

Taking into account their experience with the Web, 75% of the physicians considered that the Web has a great or very-great potential for the dissemination of guidelines and 78% indicated that they would use such guidelines if they became generally available for clinical questions that concerned them.

See Appendix 4: questions and summary of responses for questionnaire "Evaluation of guidelines."

## Discussion

Since the aim of this project was more to test the physicians' acceptance of implementing guidelines via the World Wide Web than to test the validity of the guidelines, the evaluation covered the aspects of content, form, and functioning of the Web guidelines, and acceptance by the physician. This evaluation included elements that have been identified as important in the implementation of guidelines in general [26,31]. The question of whether the guidelines led to the appropriate decision - an important question in its own right - has been and is being addressed in other studies and is not the object of this study.

To appreciate the role of the Web in the dissemination of guidelines and highlight the obstacles perceived by the physicians, several results should be emphasized:

- In spite of a highly-selected group of participants who were no doubt better equipped than the average physician in private practice, only a minority of physicians has a computer in their consultation room. Concerning the fear of disturbing the physician-patient relation, the answers are ambiguous. On the one hand, to justify the nonutilization of the guidelines for certain eligible consultations, the participants evoked fear of disturbing the relationship, and yet on the other hand, in the general evaluation at the end of the study, all participants indicated that the consultation of the guidelines involved little or no interference with that relationship.
- The user friendliness of the 2 guidelines sites was not an obstacle as the participants felt they were easy to use and that access time was acceptable. Seventy-five percent of the participants considered that the Web has great or very-great potential for the dissemination and use of guidelines.

Acceptability by the physician of implementing guidelines via the Web thus appears very high. These results allow us to conclude with confidence that the Web will be an essential tool for future guidelines-implementation strategies.

Previous studies on the possibilities of using the Web for the implementation of guidelines have shown that physician compliance is better with electronic guidelines than with paper guidelines [16]. The importance of this study in relation to previous ones is thus related to the fact that we actually tested the guidelines in clinical situations and not merely through scenarios. Our results are thus of interest in pinpointing the obstacles encountered by physicians in their daily practice.

Our study does have several limitations. Most important among these is the lack of representativity of the participants. Being a feasibility study, we chose to limit participation to physicians interested in the use of the Web in their practice and having access to a computer in their place of work. Thus, although we cannot extend our results to all physicians in Switzerland, we can reasonably consider them as representing the best-possible scenario for the implementation of guidelines via the Web, at the present time. A further limitation, also related to the nature of the study, is that we can say nothing about the appropriateness of the decisions taken by the physicians after consulting the guidelines. To have sufficient data, we asked participants to consult the guidelines even if there was no clear need to do so. This fact - consulting guidelines even if it may clearly not lead to needed information - may have affected the results.

### The Ready Availability of Guidelines on the Web is a Necessary, but Not Sufficient, Step Toward Integrating Guidelines Into Clinical Practice

The fairly-high acceptability of the Web-based guidelines needs to be tempered by the following observations:

- Only a minority of physicians have used guidelines on the Web.
- A majority of participants felt that the 2 guidelines were of little or no use in actual practice.

One of the puzzling yet important results of our study is the discrepancy between the statement that the participants wish to use the Web for guidelines and the fact that they rarely do so. The fact that the participants did not generally have ready access to a computer connected to the Internet cannot, in our opinion, explain this limited use of Web-based guidelines.

### To be Effective, Information Systems Must be Easy to Access and Use, and Must Provide Rapid Access to Appropriate Information [28]

Several hypotheses can be advanced that require further study and verifications:

- The physicians may have insufficient knowledge and awareness of medical Internet sites and not know where to find high-quality guidelines [32,8].
- The guidelines currently proposed via the Web may not correspond to the actual needs of physicians, either because of the content or because the presentations do not match up with the expectations of physicians. The transfer of paper-based guidelines to Web-based guidelines is not straightforward [13]. Guidelines need to meet certain standards and live up to certain criteria that ensure homogeneity in content and presentation [13]. The National Guideline Clearinghouse site is a step in this direction [30].
- Improved integration of guidelines into the clinical process may also facilitate use. In this vein, the integration of guidelines into computerized medical records will certainly be a fertile field of investigation [10,7]. For ready and rapid access to guidelines, a further approach that needs to be pursued, tested, and evaluated is the availability of guidelines in palm-held computing devices [33].

### Training, Standardization and Integration

In conclusion, this study demonstrated that, among motivated and fairly well-equipped physicians, the acceptability of using clinical practice guidelines via the Web is high. The main limitations to such use appear to be the absence of access to the Web at the site of consultation and perhaps the fear of the physician that consulting such guidelines will disturb the physician-patient relation. There are however numerous obstacles to overcome related to the physician or the guidelines before Web-based guidelines will become part of the everyday practice of medicine.

Future interventional studies should examine whether improved knowledge of medical Internet sites and improved skills in using those sites can increase and improve the use of Web-based clinical practice guidelines.

A minimum of standardization of Web-based guidelines might facilitate their use, and it will probably be necessary to develop and implement standardization for the quality and presentation of Web-based guidelines along the lines of what has been undertaken by the National Guideline Clearinghouse and the AGREE (Appraisal of Guidelines Research & Evaluation) collaboration [34].

Finally, it will be necessary to pursue research to better integrate the use of guidelines - in particular Web-based guidelines - into daily clinical practice.

## Multimedia Appendix 1

Screenshots of www.epage.ch (European Panel on the Appropiateness of Gastrointestinal Endoscopy)

## Multimedia Appendix 2

Computer Equipment and Previous Experience With the Web (extract)

## Multimedia Appendix 3

Use of the Guidelines Sites (extract)

## Multimedia Appendix 4

Evaluation of Guidelines (extract)
