# Supplementary material for: Use of the World Wide Web to Implement Clinical Practice Guidelines: A Feasibility Study
Source: J Med Internet Res. 2003 Jun 13;5(2):e12. doi: 10.2196/jmir.5.2.e12 (PMC1550559; doi:10.2196/jmir.5.2.e12)
Supplement: Supplementary file 1 [file jmir_v5i2e12_app1.ppt]

## Slide 1
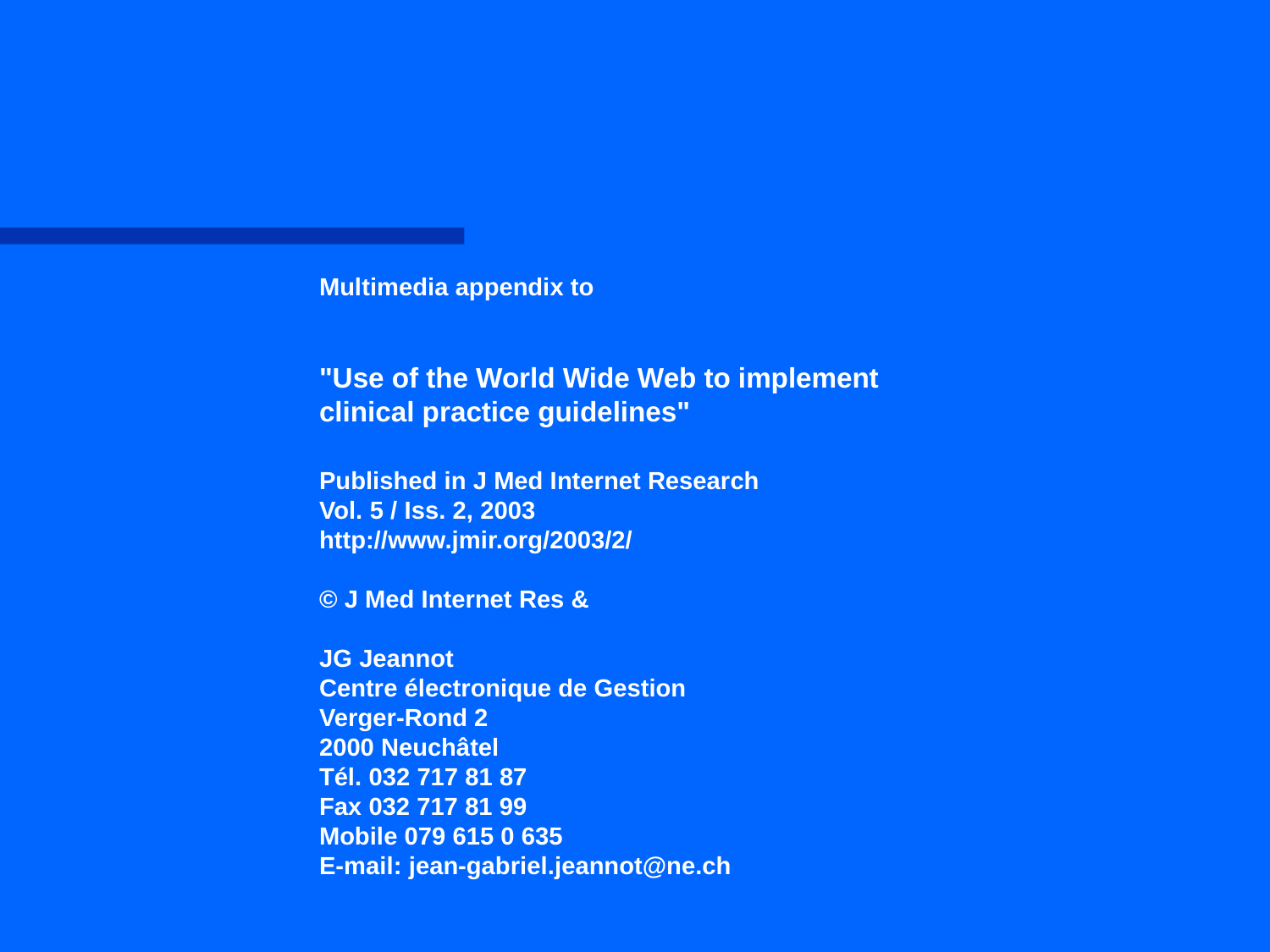

Multimedia appendix to
"Use of the World Wide Web to implement clinical practice guidelines"
Published in J Med Internet Research
Vol. 5 / Iss. 2, 2003
http://www.jmir.org/2003/2/
© J Med Internet Res &
JG Jeannot
Centre électronique de Gestion
Verger-Rond 2
2000 Neuchâtel
Tél. 032 717 81 87
Fax 032 717 81 99
Mobile 079 615 0 635
E-mail: jean-gabriel.jeannot@ne.ch

## Slide 2
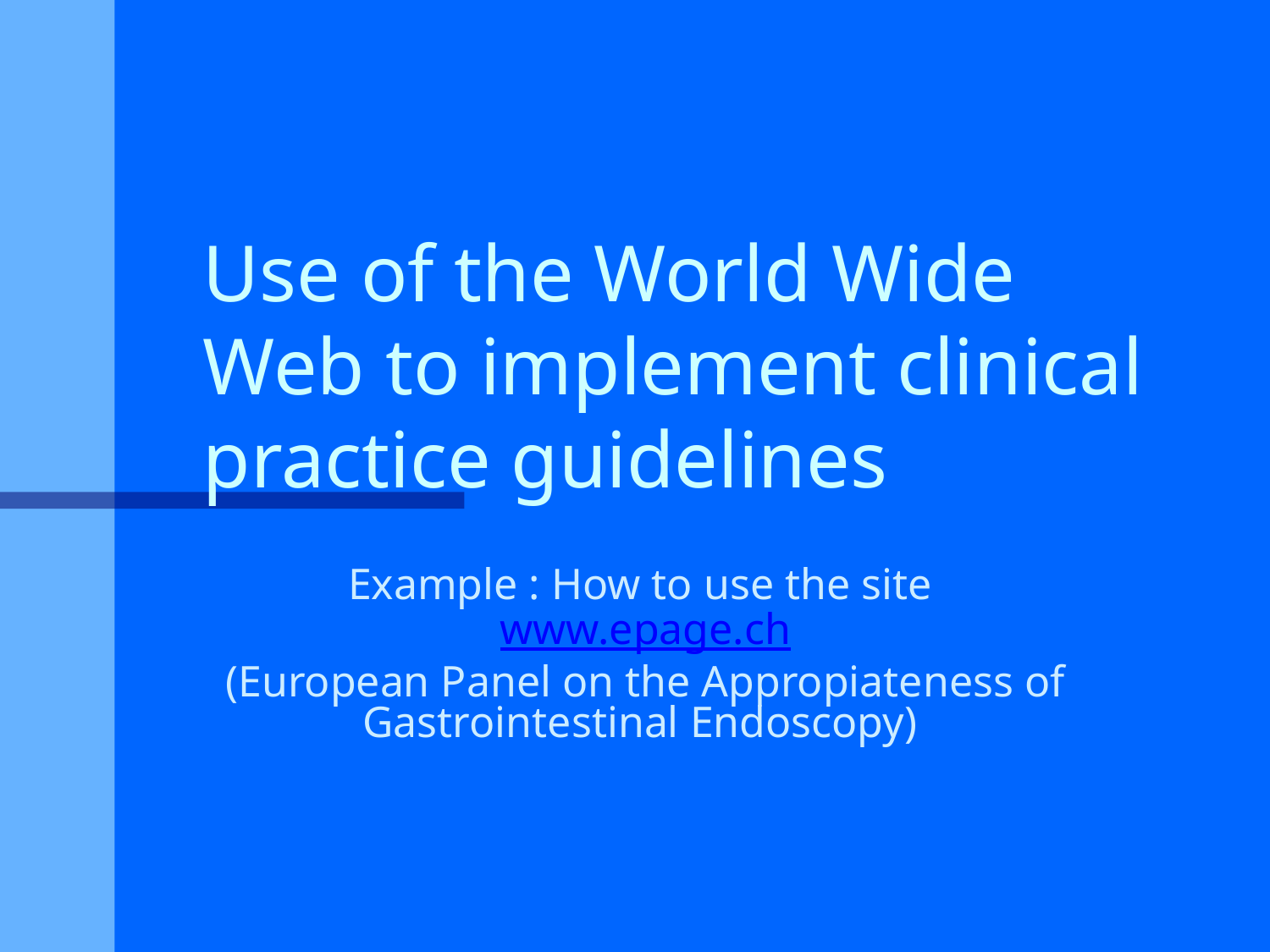

# Use of the World Wide Web to implement clinical practice guidelines
Example : How to use the site www.epage.ch
(European Panel on the Appropiateness of Gastrointestinal Endoscopy)

## Slide 3
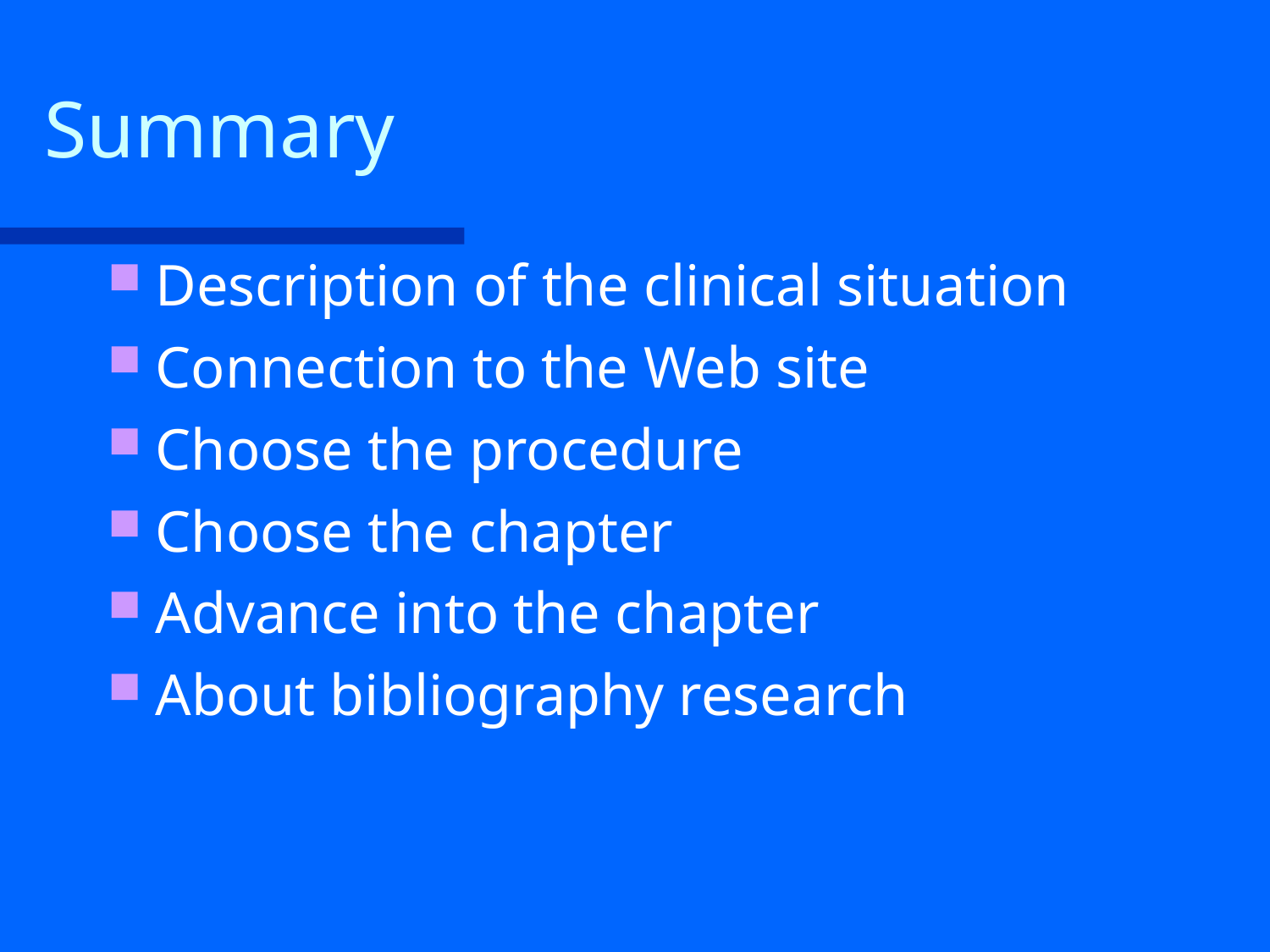

# Summary
Description of the clinical situation
Connection to the Web site
Choose the procedure
Choose the chapter
Advance into the chapter
About bibliography research

## Slide 4
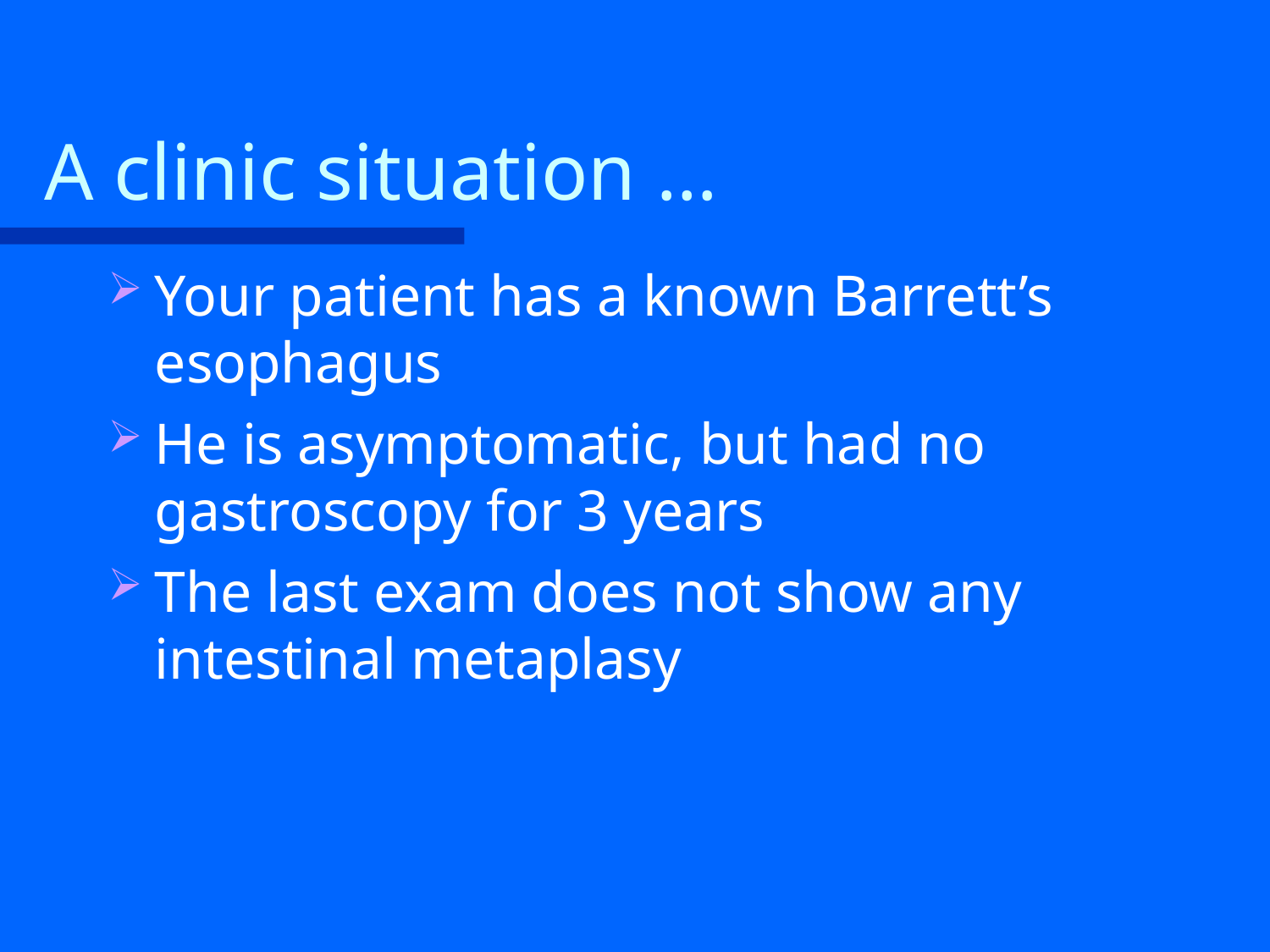

# A clinic situation …
Your patient has a known Barrett’s esophagus
He is asymptomatic, but had no gastroscopy for 3 years
The last exam does not show any intestinal metaplasy

## Slide 5
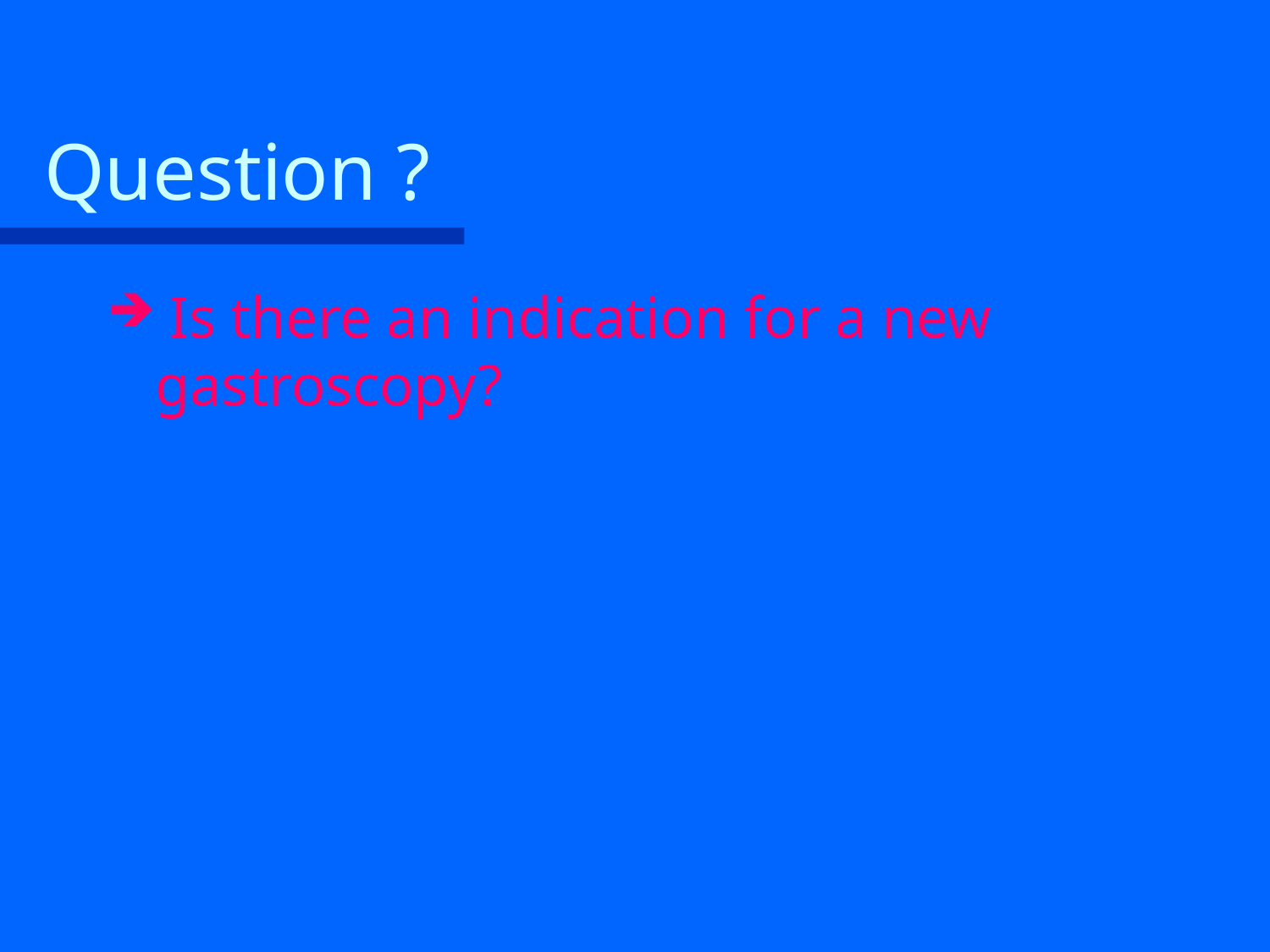

# Question ?
 Is there an indication for a new gastroscopy?

## Slide 6
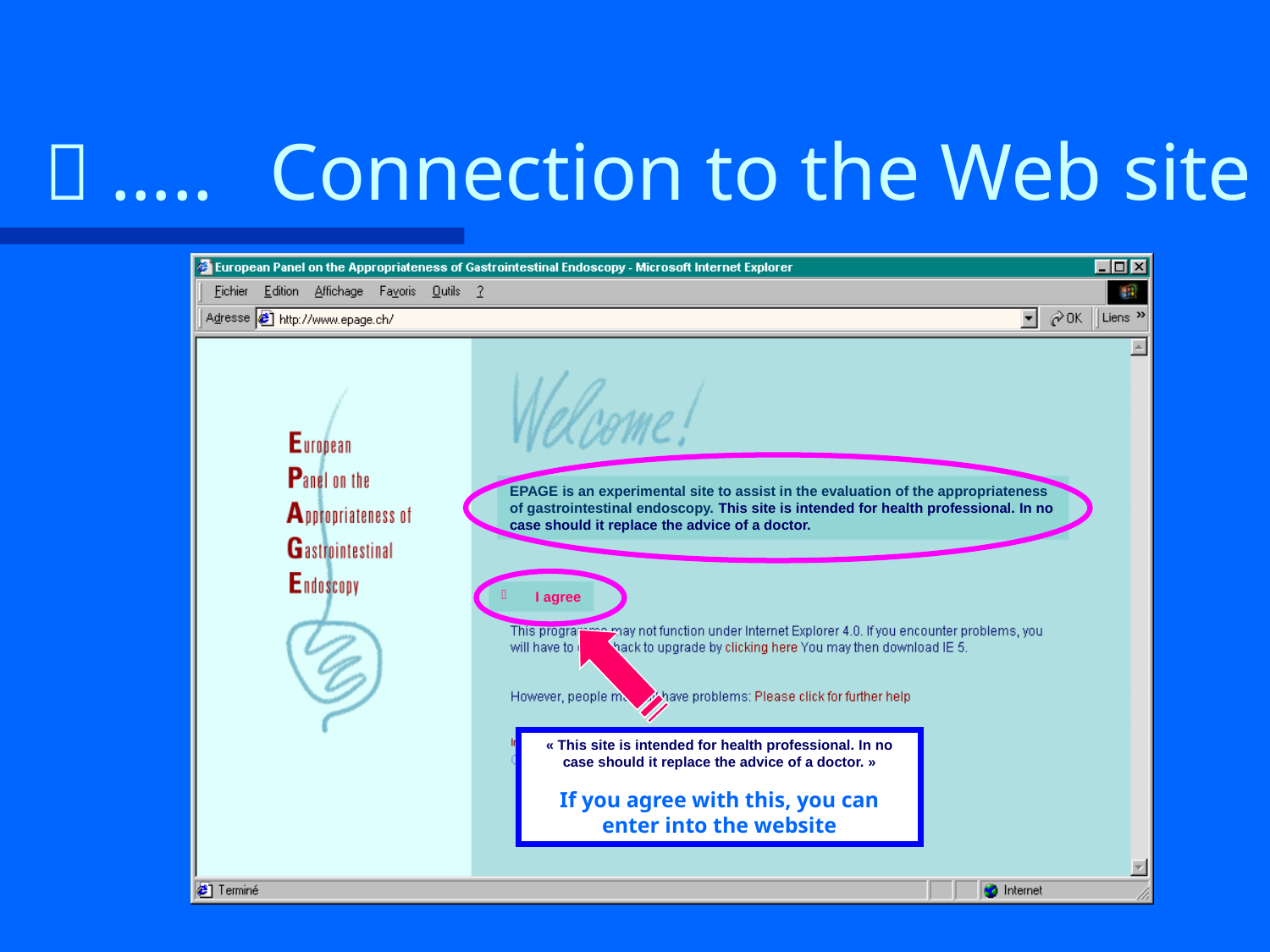

#  …..
Connection to the Web site
EPAGE is an experimental site to assist in the evaluation of the appropriateness of gastrointestinal endoscopy. This site is intended for health professional. In no case should it replace the advice of a doctor.
 I agree
« This site is intended for health professional. In no case should it replace the advice of a doctor. »
If you agree with this, you can enter into the website

## Slide 7
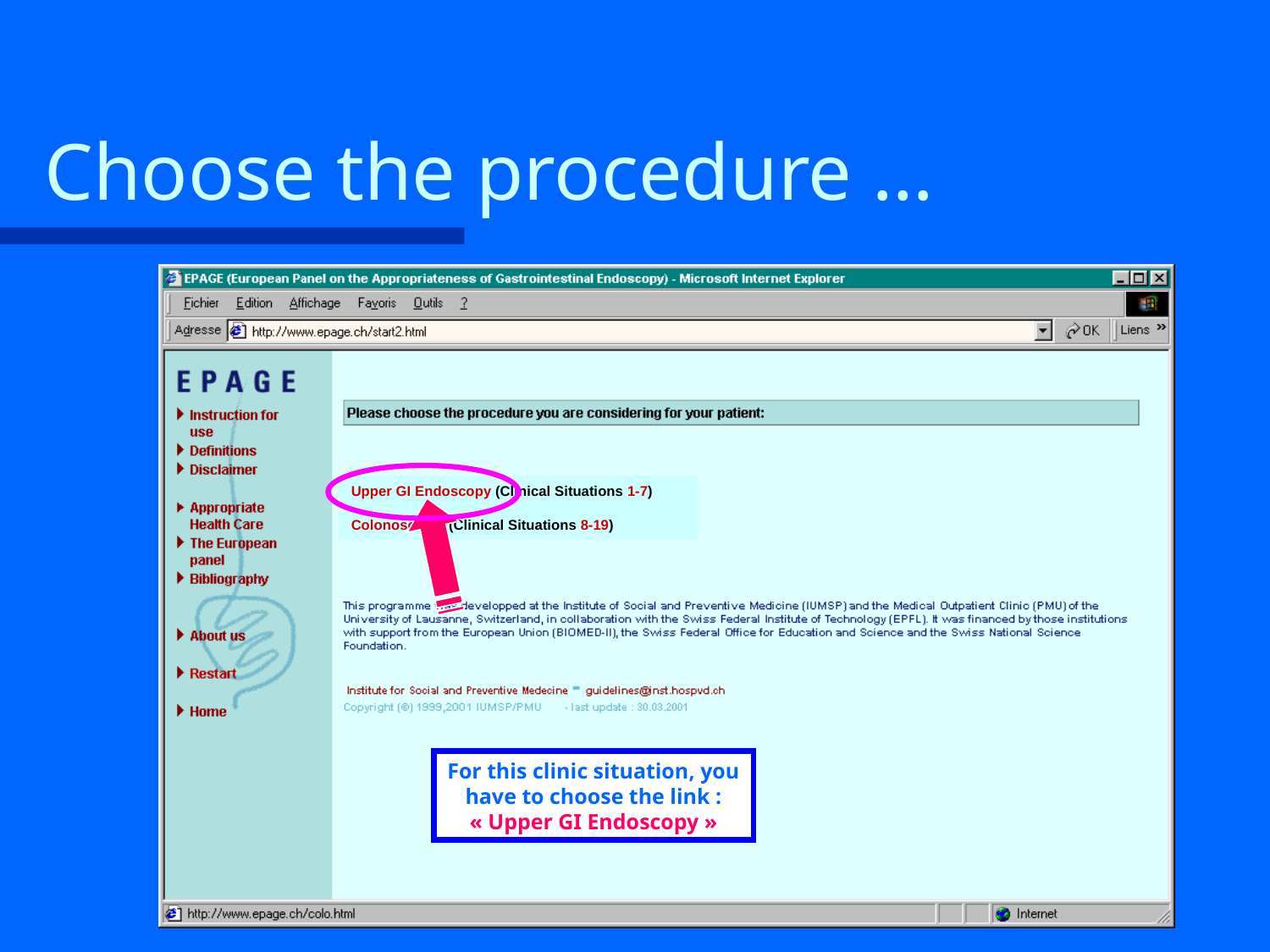

# Choose the procedure …
Upper GI Endoscopy (Clinical Situations 1-7)
Colonoscopy (Clinical Situations 8-19)
For this clinic situation, you have to choose the link :
« Upper GI Endoscopy »

## Slide 8
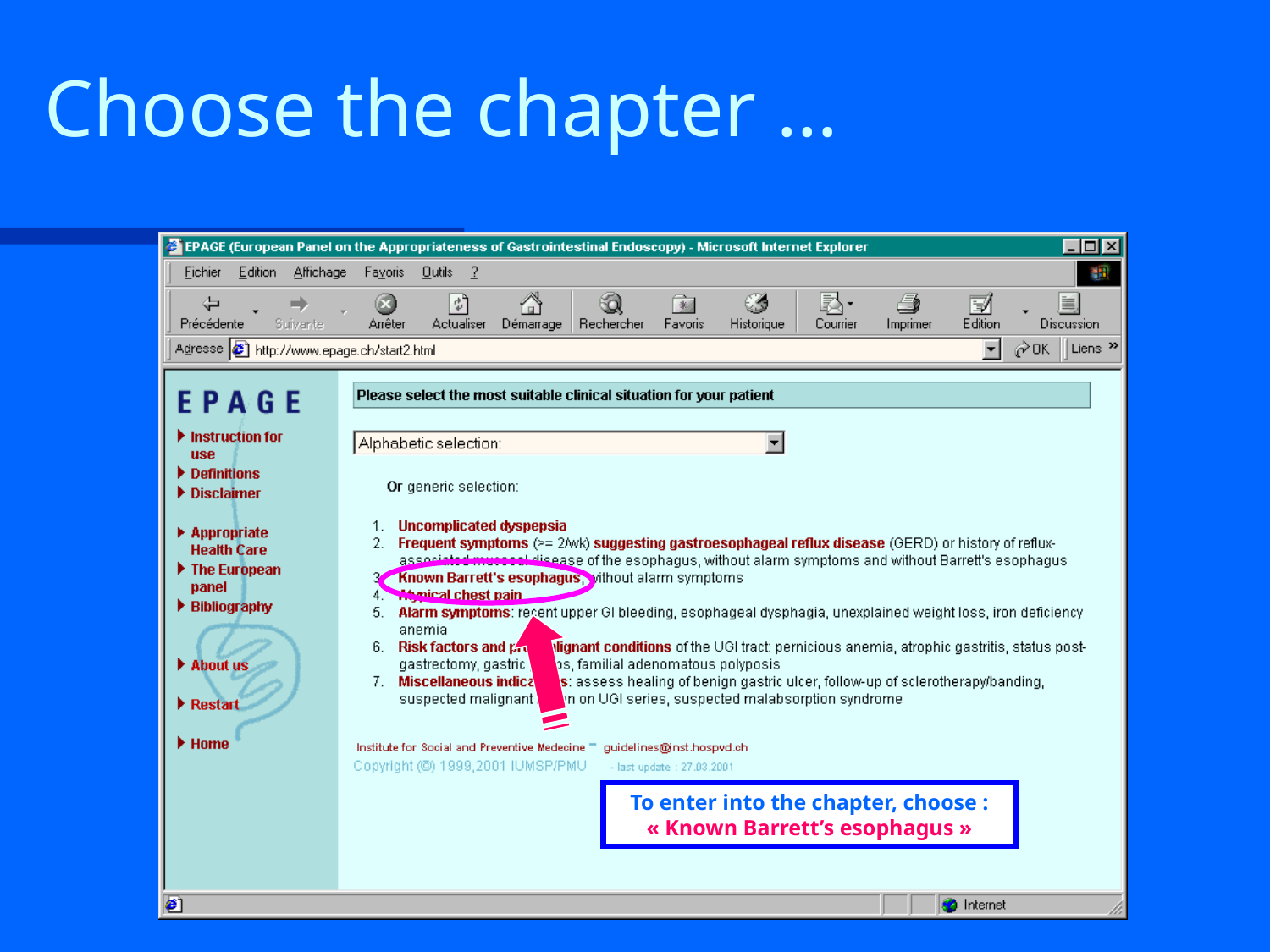

# Choose the chapter …
To enter into the chapter, choose :
« Known Barrett’s esophagus »

## Slide 9
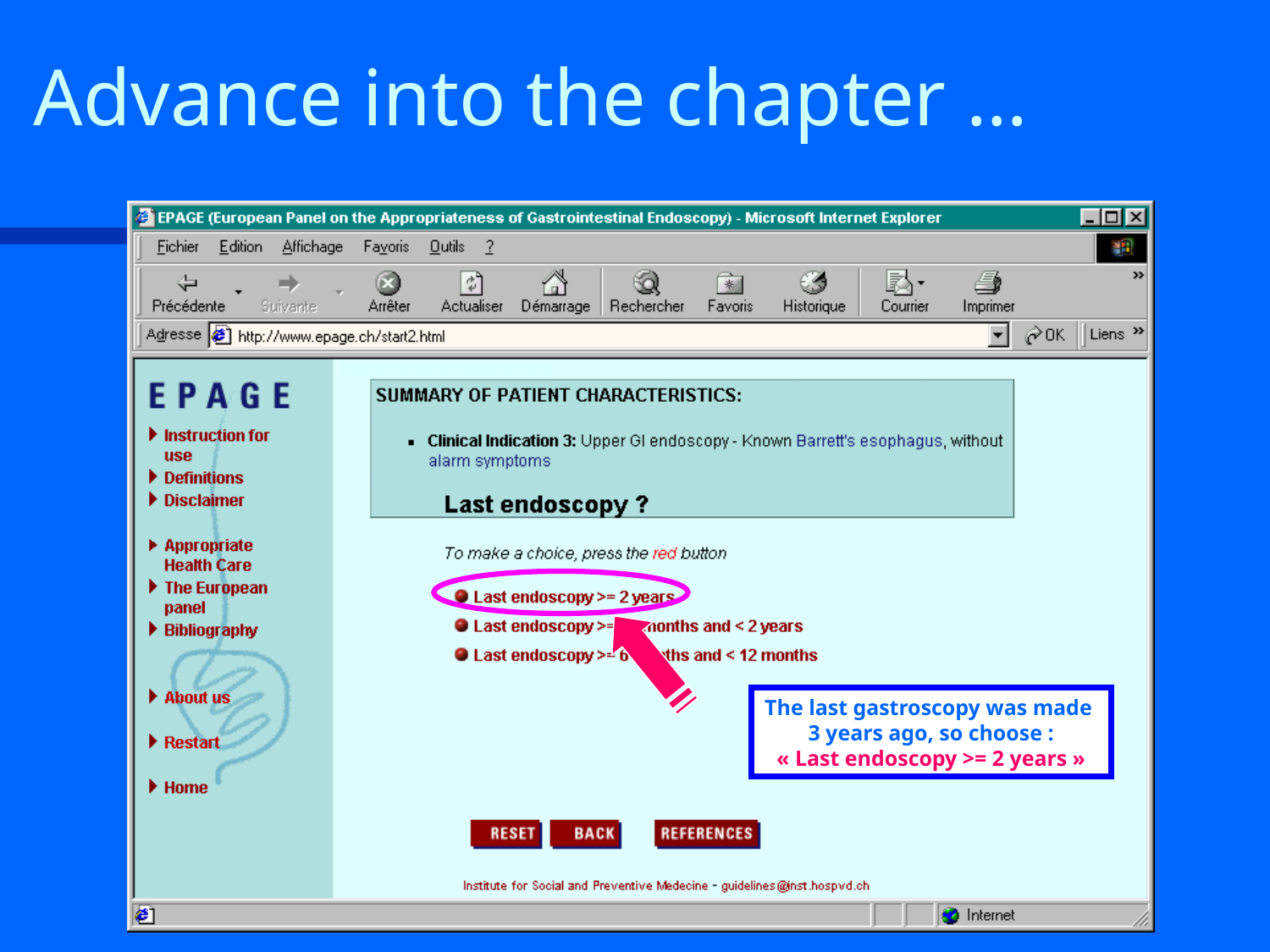

# Advance into the chapter …
The last gastroscopy was made 3 years ago, so choose :
« Last endoscopy >= 2 years »

## Slide 10
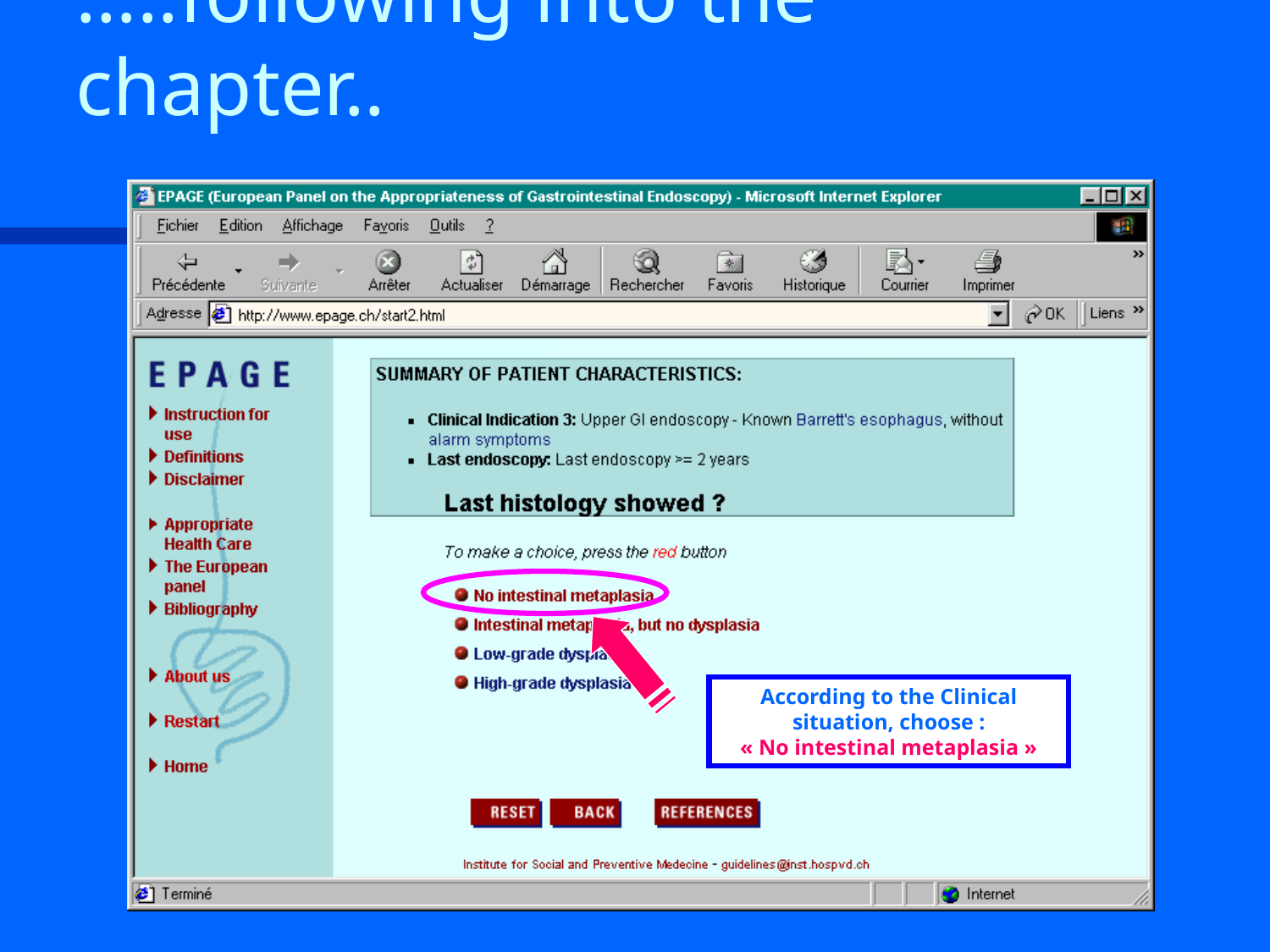

# …..following into the chapter..
According to the Clinical situation, choose :
« No intestinal metaplasia »

## Slide 11
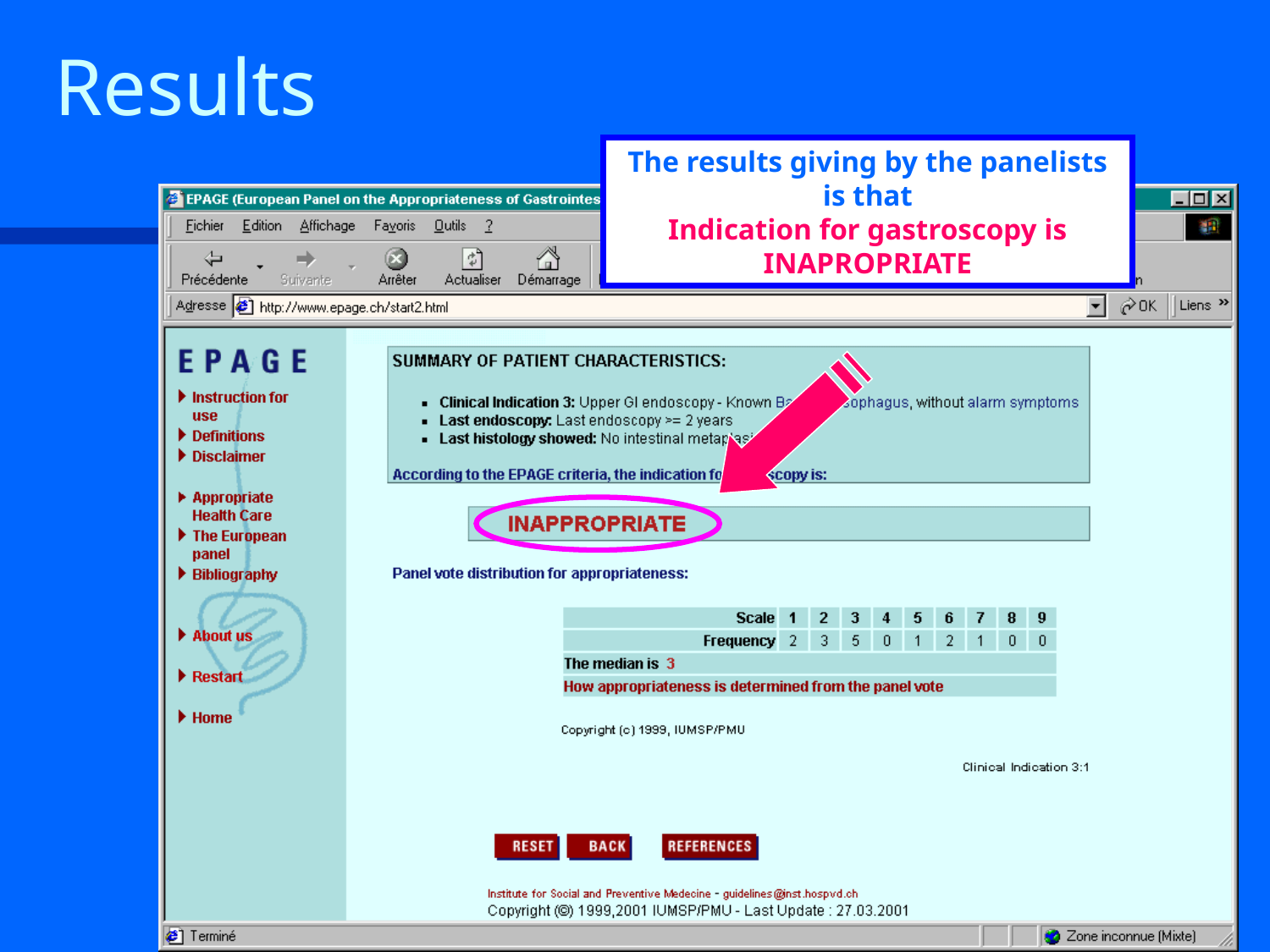

# Results
The results giving by the panelists is that
Indication for gastroscopy is INAPROPRIATE

## Slide 12
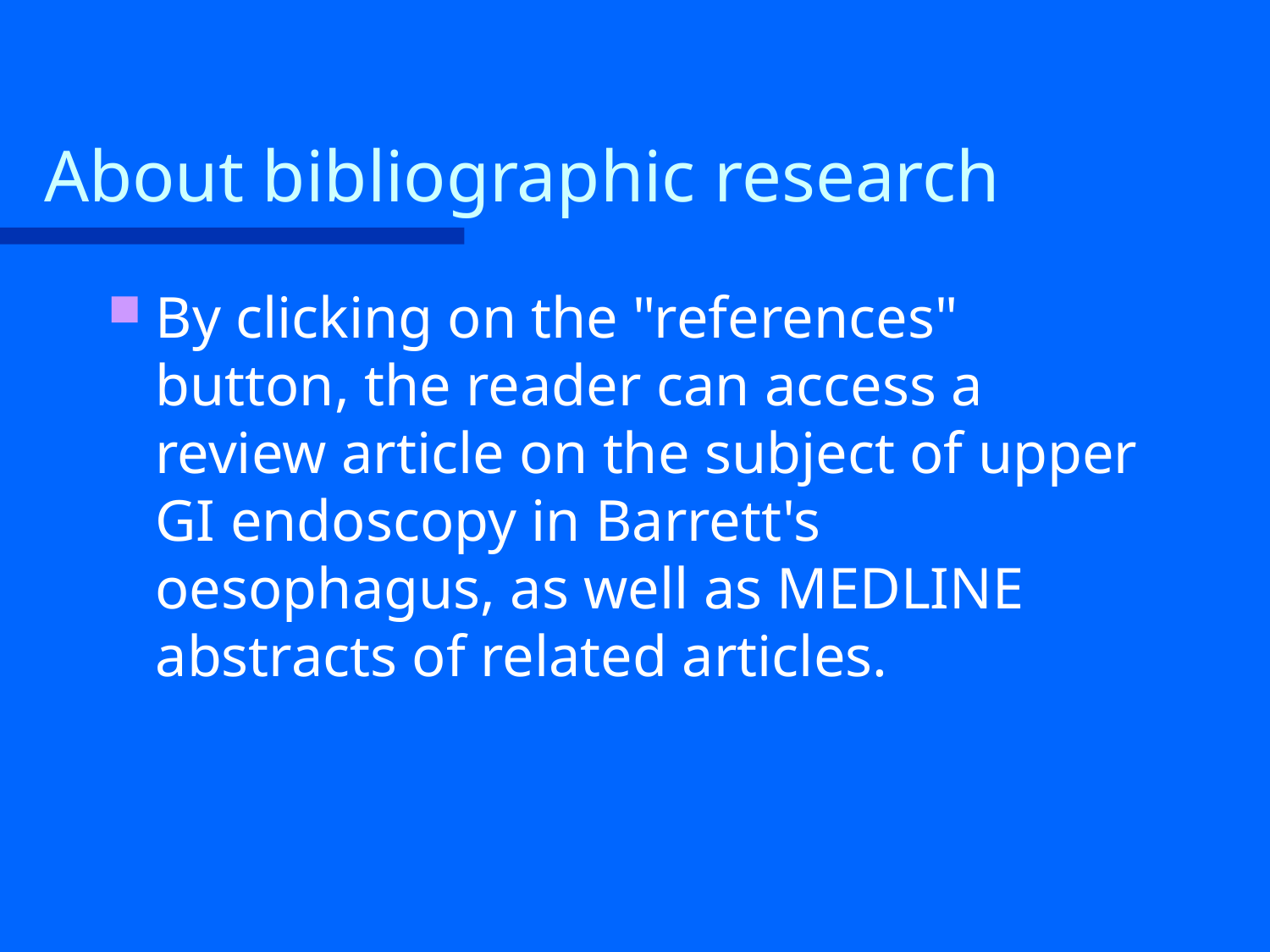

# About bibliographic research
By clicking on the "references" button, the reader can access a review article on the subject of upper GI endoscopy in Barrett's oesophagus, as well as MEDLINE abstracts of related articles.

## Slide 13
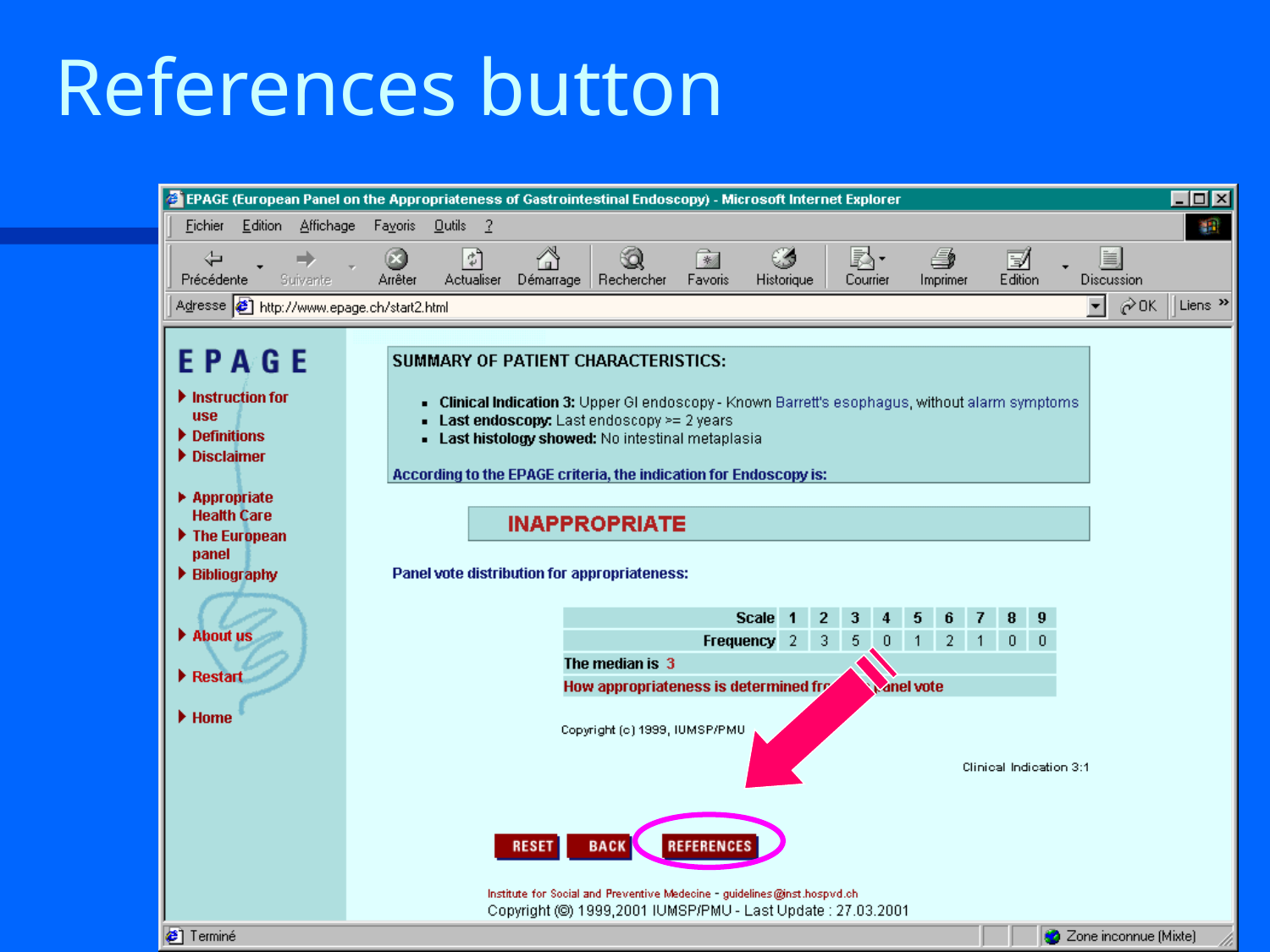

# References button
