# Supplementary material for: Use of the World Wide Web to Implement Clinical Practice Guidelines: A Feasibility Study
Source: J Med Internet Res. 2003 Jun 13;5(2):e12. doi: 10.2196/jmir.5.2.e12 (PMC1550559; doi:10.2196/jmir.5.2.e12)
Supplement: Supplementary file 2 [file jmir_v5i2e12_app2.html]

Nouvelle page 1

**### Computer equipment and previous experience with the web (extract)**


---

**How often do you feel guidelines (in general, not those from the web) are
useful in deciding on patient care?**

- very often: 2
- sometimes: 17
- rarely: 1
- never: 0

**Have you had the occasion to use other guidelines from the WWW?**

- Yes: 3
- No: 16

**If so, please indicate the name of the web site or the internet address?**

- Association médicale canadienne: http://www.cma.ca/cpgs/index.asp
- Université de Californie: http://www.ucsf.edu
- Société britannique de l'hypertension: http://www.hyp.ac.uk/bhs/home.htm

**Please indicate what kind of computer you use?**

- PC: 16
- Mac: 5
- Other: 0

**Size of the screen:**

- 13": 1
- 15": 10
- 17": 5
- 19": 1
- Bigger: 1
- Other: 2

**Do you ever use the Internet?**

- Yes: 20
- No: 0

**Do you use the WWW regularly (at least once/week)?**

- Yes: 18
- No: 2

**Is your use of the WWW mainly to access medical sites or non-medical sites?**

- Mainly medical sites: 4
- Both medical and non-medical sites: 13
- Mainly non-medical sites: 2

**Do you have more than one computer in your office?**

- Yes: 17
- No: 2

**How did you usually access the internet when using the EPAGE site ?**

- Modem (analog): 8
  - 14.4 KB/s: 0
  - 28.8 KB/s: 0
  - 56 KB/s: 8
  - Other: 0
- ISDN: 8
- ADSL: 0
- Cable: 3
- Other: 1
