# Supplementary material for: Use of the World Wide Web to Implement Clinical Practice Guidelines: A Feasibility Study
Source: J Med Internet Res. 2003 Jun 13;5(2):e12. doi: 10.2196/jmir.5.2.e12 (PMC1550559; doi:10.2196/jmir.5.2.e12)
Supplement: Supplementary file 3 [file jmir_v5i2e12_app3.html]

Use of the guidelines sites


**### Use of the guidelines sites (extracts)**

---

**Patient sex**

- Male: 95
- Female: 118

**Did you decide to consult the guidelines for this patient?**

- Yes: 153
- No: 53

**If not, please indicate the reason**

- No time: 11
- Computer unavailable: 5
- Would have interfered with patient-physician relationship: 27
- Other: 22 (generally physicians who stated that the clinical situation was
  sufficiently clear that no additional information could be expected from
  consulting the guidelines.)

**Were you able to access the epage site ?**

- Yes: 159
- No: 7

**Which guidelines did you consult ?**

- Gastroscopy: 45
- Colonoscopy: 35
- Laminectomy: 104

**How long did the actual access of the epage web site last? (history +
clinical examination + consultation of guidelines)**

- Average: 27 minutes.

**How long did the consultation of the guidelines itself last ?**

- Average 3.4 minutes or 12 % of total consultation time.

**Was the computer turned on when you wanted to access the site ?**

- Yes: 159
- No: 29

**If you use a dial-in modem : Was the connection established without difficulty ?**

- Yes: 141
- No: 8

**Were you able to reply to the proposed choices on the epage web site without difficulty ?**

- Yes: 168
- No: 7

**What was the proposed procedure ?**

- Appropriate: 56
- Uncertain: 24
- Inappropriate: 95

**Do you agree with the proposed appropriateness ?**

- Yes: 163
- No: 18

**Did consulting guidelines influence your choice of treatment for the patient ?**

- Yes: 14
- No: 169

**Did you discuss different treatment modalities with this patient ?**

- Yes: 145
- No: 41

**If so, did the patient accept your proposal ?**

- Yes: 138
- No: 8

**Were the guidelines of assistance to you in your handling of this patient ?**

- Yes: 15
- No: 169
