# Supplementary material for: Use of the World Wide Web to Implement Clinical Practice Guidelines: A Feasibility Study
Source: J Med Internet Res. 2003 Jun 13;5(2):e12. doi: 10.2196/jmir.5.2.e12 (PMC1550559; doi:10.2196/jmir.5.2.e12)
Supplement: Supplementary file 4 [file jmir_v5i2e12_app4.html]

Evaluation of guidelines


**### Evaluation of guidelines (extract)**


---

**How would you characterize, in general, the ease of access to the EPAGE
guidelines Web site for "Laminectomy" ?**

From very easy (1) to extremely frustrating (6)

|  |  |  |  |  |  |
| --- | --- | --- | --- | --- | --- |
| **1** | **2** | **3** | **4** | **5** | **6** |
| 12 | 8 | 0 | 0 | 0 | 0 |

- I didn't use the site: 0

**How would you characterize, in general, the ease of access to the EPAGE
guidelines Web site for "Endoscopy" ?**

From very easy (1) to extremely frustrating (6)

|  |  |  |  |  |  |
| --- | --- | --- | --- | --- | --- |
| **1** | **2** | **3** | **4** | **5** | **6** |
| 4 | 10 | 0 | 0 | 0 | 1 |

- I didn't use the site: 0

**If you did use both sites, which one did you prefer ?**

- Endoscopy: 7
- Laminectomy: 3
- Both are equivalent: 3

**In general, was the time required to access the guidelines acceptable ?**

- Yes: 19
- No: 1

If not, what would be an acceptable delay ?

- 1 response: 10 seconds.

**If you did not use the guidelines with all eligible patients, what are the
major reasons (more than one response possible) ?**

- Computer not in examination room: 3
- Computer not ready (computer not turned on OR Web-browser not loaded OR Web
  site not accessed): 2
- Felt patient would not agree: 0
- Too busy: 9
- Situation was clear. No need for guidelines: 8
- Others: 4

**Given your experience with the EPAGE guidelines, how would
you rate the *current ease of use* in terms of making them a feasible tool
to assist in medical decisions?**

From 1 (very easy to use) to 6 (extremely difficult to use)

|  |  |  |  |  |  |
| --- | --- | --- | --- | --- | --- |
| **1** | **2** | **3** | **4** | **5** | **6** |
| 4 | 8 | 3 | 2 | 2 | 1 |

**Given your experience with the EPAGE guidelines, how would
you rate their *current usefulness* as an aid in determining the
appropriateness of medical procedures?**

From 1 (very useful) to 6 (totally useless)

|  |  |  |  |  |  |
| --- | --- | --- | --- | --- | --- |
| **1** | **2** | **3** | **4** | **5** | **6** |
| 1 | 3 | 2 | 10 | 3 | 1 |

**How would you rate the learning experience from the use of
the EPAGE guidelines?**

From 1 (enriching) to 6 disappointing).

|  |  |  |  |  |  |
| --- | --- | --- | --- | --- | --- |
| **1** | **2** | **3** | **4** | **5** | **6** |
| 0 | 3 | 5 | 3 | 6 | 3 |

**Given your experience using the WWW (not only with the EPAGE
site), how would you rate its potential to vehicle clinical practice guidelines?**

From 1 (tremendous potential) to 6 (none).

|  |  |  |  |  |  |
| --- | --- | --- | --- | --- | --- |
| **1** | **2** | **3** | **4** | **5** | **6** |
| 6 | 4 | 5 | 3 | 1 | 0 |

**How much did use of the EPAGE guidelines *disturb you*
in *your* relation to your patients?**

From 1 (not at all) to 6 (very much).

|  |  |  |  |  |  |
| --- | --- | --- | --- | --- | --- |
| **1** | **2** | **3** | **4** | **5** | **6** |
| 10 | 5 | 1 | 1 | 0 | 0 |

**How much do you feel your use of /reference to the EPAGE
guidelines *disturbed the patient*?**

From 1 (not at all) to 6 (very much).

|  |  |  |  |  |  |
| --- | --- | --- | --- | --- | --- |
| **1** | **2** | **3** | **4** | **5** | **6** |
| 4 | 9 | 1 | 0 | 0 | 0 |

**How much did this *disturb you* in your established
pattern of work?**

From 1 (not at all) to 6 (very much).

|  |  |  |  |  |  |
| --- | --- | --- | --- | --- | --- |
| **1** | **2** | **3** | **4** | **5** | **6** |
| 3 | 5 | 6 | 4 | 1 | 0 |

**Do you plan to use the EPAGE guidelines beyond the study period ?**

- Yes: 14
- No: 4

**If no, please indicate the reason (indicate all that apply):**

- Access to network too slow : 0
- Program itself too difficult to use : 0
- Program did not function well : 1
- Time and effort not worth information received : 4
- Subjects for guidelines in this program not well chosen : 1
- I disagree with the recommendations of the guidelines : 0
- I generally don't need practice guidelines : 3
